# CB‐103: A novel CSL‐NICD inhibitor for the treatment of NOTCH‐driven T‐cell acute lymphoblastic leukemia: A case report of complete clinical response in a patient with relapsed and refractory T‐ALL

**DOI:** 10.1002/jha2.510

**Published:** 2022-06-16

**Authors:** Michael Medinger, Till Junker, Dominik Heim, Alexandar Tzankov, Philip M. Jermann, Maria Bobadilla, Michele Vigolo, Rajwinder Lehal, Florian D. Vogl, Michael Bauer, Jakob Passweg

**Affiliations:** ^1^ Department of Hematology University Hospital Basel Basel Switzerland; ^2^ University Basel Basel Switzerland; ^3^ Institute of Medical Genetics and Pathology University Hospital Basel Basel Switzerland; ^4^ Cellestia Biotech AG Basel Switzerland

**Keywords:** acute lymphoblastic leukaemia, allogeneic hematopoietic stem cell transplantation, NOTCH inhibition

## Abstract

Relapsed T cell acute lymphoblastic leukaemia (T‐ALL) has a very poor prognosis. A 24‐year‐old patient with relapsed high‐risk T‐ALL (PTEN gene deletion; NOTCH1 mutation), was treated with the NOTCH inhibitor CB‐103. Within 1 week of starting CB‐103, the bone marrow was free of T‐ALL blast infiltration (MRD+) and successfully underwent allogeneic hematopoietic stem cell transplantation (allo‐HSCT). Sequential samples of ctDNA to monitor the disease after allo‐HSCT showed a decrease of circulating Notch1 and PTEN alterations. This is the first T‐ALL patient treated with CB‐103. The observed clinical response encourages further exploration of CB‐103 in ALL.

## 1 INTRODUCTION

NOTCH signaling is a highly conserved pathway, performing essential functions including specification of cell fate, proliferation, and apoptosis [1]. Aberrant activation of the NOTCH pathway plays a key role in tumor initiation, maintenance, and resistance to cancer therapy [2, 3]. NOTCH appears as a recurrently mutated oncogene in lymphoproliferative disorders, including T‐cell acute lymphoblastic leukemia (T‐ALL) [1]. Approximately 60% of T‐ALL cases have NOTCH pathway activation due to mutations in NOTCH  and/or FBXW7 genes [4, 5, 6, 7]. Furthermore, multiple NOTCH aberrations may develop during disease progression, resulting in a more aggressive phenotype [8].

Activation of the NOTCH pathway through NOTCH ligands and receptors interaction leads to dissociation of the intracellular domain of these NOTCH receptors (NICD), which enter the nucleus and bind to a DNA‐bound repressor complex that includes CSL (CBF in vertebrates, Suppressor of Hairless in Drosophila melanogaster, Lag‐1 in Caenorhabditis elegans). This results in a transcriptional activation that mediates downstream pro‐tumorigenic effects [1, 9, 10]. However, despite the frequent NOTCH pathway activation, none of the drugs approved for T‐ALL treatment specifically target NOTCH [11].

CB‐103 is a highly selective and potent inhibitor of the CSL‐NICD gene transcription complex, acting as a pan‐NOTCH inhibitor. Preclinical studies have demonstrated that CB‐103‐mediated inhibition of CSL‐NICD complex circumvents dose‐limiting toxicities associated with previous generations of NOTCH inhibitors, i.e., Gamma Secretase Inhibitors (GSIs) and monoclonal antibodies against NOTCH ligands and receptors (MABs) [2]. CB‐103 displayed an excellent safety profile in a Phase 1 dose‐escalation study in patients with solid tumors [12], notably with absence of toxicities typically associated with GSIs and MABs. Treatment with CB‐103 as a single agent in patients with NOTCH‐activated, metastatic adenoid cystic carcinoma resulted in cases with tumor regression, stop of growth, and long‐term disease control in patients with documented PD before treatment. Median PFS was 21.7 weeks, representing a clear clinical benefit in this patient population with fast‐progressing tumors. NOTCH pathway biomarkers in surrogate tissues showed strong target engagement [12].

## 2 CASE

Here we describe the case of a patient with relapsed/refractory (r/r) T‐ALL, who achieved complete remission (CR) and became minimal residual disease (MRD)‐negative after addition of CB‐103 treatment to ongoing therapy. The patient provided written informed consent before receiving experimental therapy with CB‐103 and for the reporting of his case.

The affected individual was a 24‐year‐old male diagnosed with T‐ALL harboring an L1678P activating mutation of NOTCH1 with a variant allelic frequency (VAF) of 29%, assessed in a bone marrow sample with a 95% blast infiltration, which would speak in favour of a clonal mutation.

After achieving an initial CR with induction and consolidation therapy, the patient relapsed within a few months and became refractory to a series of salvage therapies (Supplementary Table 1). When all standard treatments were exhausted, a sequential/combined therapy with agents specifically targeted to the molecular alterations detected in his leukemic cells (BCL2, ABL1, CD38, and NOTCH1) was adopted as a final attempt at treatment before transitioning the patient to allogeneic hematopoietic stem cell transplantation (allo‐HSCT).

**FIGURE 1:**
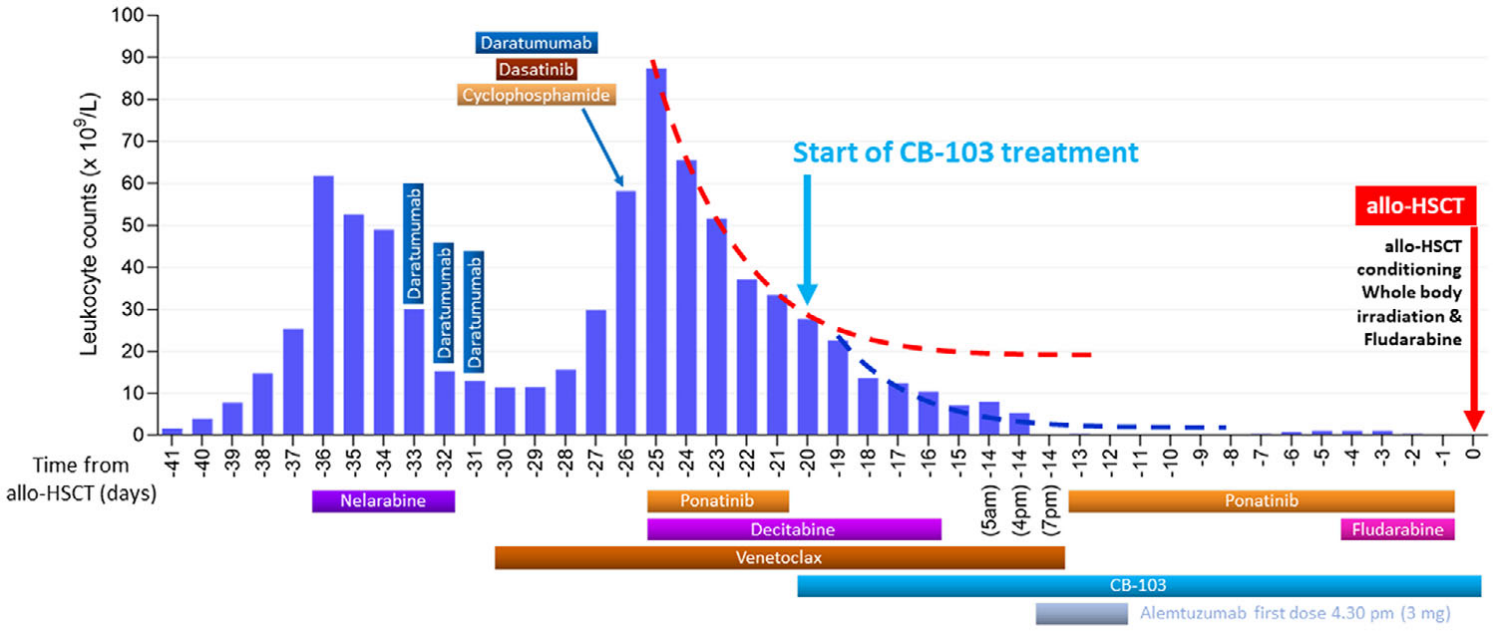
Leukocyte counts and anti‐neoplastic therapies during the days before allogeneic Hematopoietic Stem Cell Transplantation (allo‐HSCT). Each bar displays peripheral blood leukocyte counts on the indicated days before allogeneic hematopoietic stem cell transplantation (allo‐HSCT). Treatment intervals are described with the horizontal bars. In details: Nelarabine (day ‐36 to day ‐32); Ponatinib (day ‐25 to day ‐21 and day ‐13 to day ‐01); Decitabine (day ‐25 to day ‐16); Venetoclax (day ‐30 to day ‐14); CB‐103 (day ‐20 to day +28); Alemtuzumab (day ‐14 at 4:30 pm to day ‐12); Fludarabine (day ‐4 to day ‐1)

**FIGURE 2:**
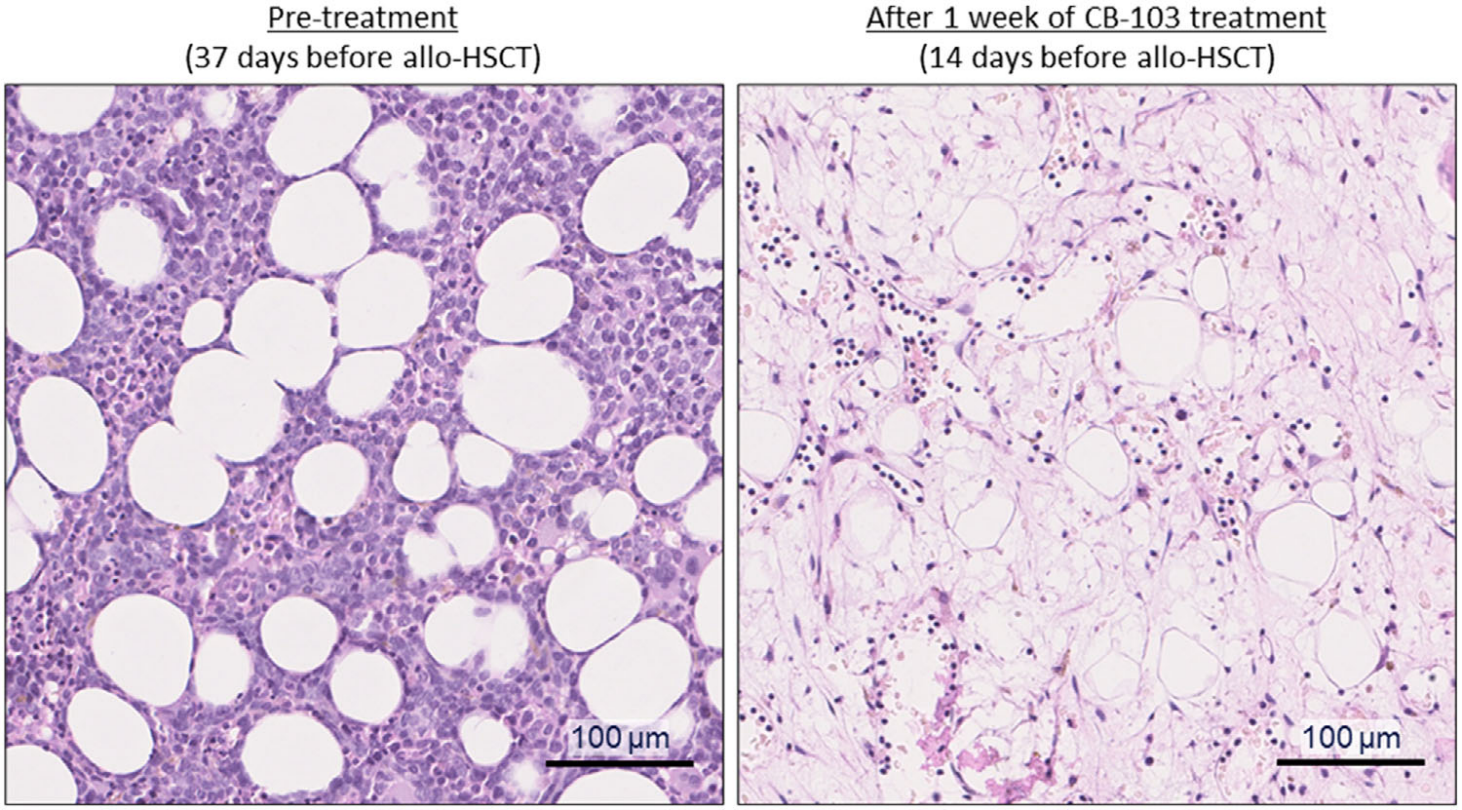
Bone marrow status before and after CB‐103 treatment. Hematoxylin and eosin staining of bone marrow biopsy obtained 37 days before stem cell transplantation (SCT) (left) and after one week of CB‐103 treatment but before alemtuzumab administration (right)

After an initial partial response to treatment with venetoclax, ponatinib, and decitabine, the patient's response to treatment began to wane. This, in addition to tolerability issues, led to the step‐wise reduction of this triple combination. At this time, the persistence of the pathogenic NOTCH1 mutation with a VAF of 33% was confirmed in a bone marrow biopsy. Under a compassionate use protocol, the patient received CB‐103 (Cellestia Biotech AG, Basel, Switzerland). Using a rapid dose‐escalation schedule, CB‐103 was added to the treatment with venetoclax and decitabine, both of which were gradually phased out while the daily dose of CB‐103 was increased. Figure 1 illustrates the timeline of leukocyte counts and anti‐neoplastic therapies during the weeks preceding allo‐HSCT. RNA was extracted from circulating blasts before and one hour after the first dose of CB‐103 for gene expression profiling (performed using NanoString technology) [13]. This analysis showed downregulation of NOTCH target genes, including Cyclin D3, Deltex‐1 (DTX1) and NOTCH1 (Figure S1). Bone marrow biopsy after seven days on CB‐103 treatment revealed clearance of the blasts (Figure 2).

The downregulation of NOTCH target genes together with a change in the slope of the leukocyte count decrease curve, and the blast clearance in bone marrow in this patient with an extremely poor prognosis at the time of CB‐103 treatment initiation, suggest a contribution of CB‐103 to disease control.

As the circulating T‐ALL population was CD52‐positive, alemtuzumab was initiated as an additional treatment but discontinued given the limited effects observed on the target cell population. Yet, the patient remained in remission and was able to undergo allo‐HSCT two weeks later. The conditioning regimen was fludarabine 60 mg (30 mg/m^2^) day ‐4 to day ‐1 and total body irradiation (8 Gy) and graft‐versus‐host disease (GvHD) prophylaxis with cyclosporine A. CB‐103 was maintained throughout the conditioning, transplantation, and post‐allo‐HSCT period to control the clone harboring the NOTCH1 mutation. Ponatinib was also administered post‐allo‐HSCT since the patient had tested positive to NUP214(32)‐ABL1(2) fusion before allo‐HSCT.

Variations in circulating tumor DNA (ctDNA) levels were analyzed in liquid biopsies obtained before and after the patient underwent allo‐HSCT. Next‐generation sequencing (NGS) of the ctDNA indicated the presence of T‐ALL gene variants (ARID1A D1462E, FGFR1 R507C, NOTCH1 L1678P, and PTEN L25S), indicative of residual disease before allo‐HSCT. The VAF of the individual variants was between 38–73% two weeks before allo‐HSCT, and fell to <8% on the date of allo‐HSCT. Two weeks after allo‐HSCT, under continued CB‐103 therapy, variants were no longer detectable in ctDNA (assay sensitivity 0.5‐1.0%), suggestive of disease control. On day +87 the patient achieved a molecular CR, testing negative for the T‐cell receptor rearrangement V1JJD1 and Vb19Jb1.6 (as evaluated using reverse transcription‐quantitative real‐time PCR) (Figure S2 and Supplement 1_ Methods). The patient remained disease‐free for six months. He subsequently developed an aggressive disseminated extramedullary relapse (including lungs, skin, intestine), for which targeted treatment was started (Ponatinib, idelalisib, CB‐103, dexamethasone, daratumumab). The patient eventually died of progressive extramedullary disease on day +259 after HSCT.

This case report describes a r/r T‐ALL patient who received CB‐103. Addition of CB‐103 to a salvage therapy achieved CR within one week of treatment initiation, and continued treatment with CB‐103 during and after allo‐HSCT contributed to MRD eradication and sustained disease control. CB‐103 was well‐tolerated in combination with other anti‐cancer agents (including venetoclax, decitabine, and alemtuzumab) and total body irradiation. Adverse events deemed related to CB‐103 were mild, grade 1 visual disturbances and nausea. Within one week of starting CB‐103 treatment, the bone marrow was free of T‐ALL blast infiltration, enabling the patient to undergo allo‐HSCT. The encouraging clinical response obtained with CB‐103 in this indication, in a patient that had exhausted all other available options and dismal prospect for survival, warrants further investigation. A clinical trial is currently underway (NCT03422679).

## CONFLICT OF INTEREST

The study was supported by Cellestia Biotech AG, Basel, Switzerland. MBo, MV, RL, FDV, and MB are employees of Cellestia Biotech AG, Basel, Switzerland. The other authors declare no competing interest.

## ETHICAL STATEMENT

The study was performed according to the regulations of the local ethics committee. The patient has consented to participate and the use of materials.

## AUTHOR CONTRIBUTIONS

Michael Medinger, Jakob Passweg, Till Junker, Joerg Halter, and Dominik Heim performed the research. Michael Medinger, Jakob Passweg, Florian D. Vogl, and Michael Bauer, designed the research study. Philip M. Jermann, Alexandar Tzankov, Maria Bobadilla, Rajwinder Lehal, and Michele Vigolo contributed essential reagents or tools. Michael Medinger, Philip M. Jermann, Alexandar Tzankov, Florian D. Vogl, Maria Bobadilla, Michele Vigolo, and Rajwinder Lehal analyzed the data. Michael Medinger, Florian D. Vogl, Maria Bobadilla, and Michele Vigolo wrote the manuscript.

## Supporting information

Supplement 1_ Methods

Supplementary Figure 1 – NOTCH pathway target‐gene downregulation 1 hour after CB‐103 administration. Gene expression profiling in peripheral blasts performed using NanoString technology showed downregulation of NOTCH target genes, including Cyclin D3, Deltex‐1 and NOTCH1.

Figure S2 ‐ Presence of T‐ALL molecular markers in liquid biopsies following allogeneic Hematopoietic Stem Cell Transplantation (allo‐HSCT). Next‐generation sequencing of circulating tumour DNA was performed at various timepoints to follow up the identified T‐ALL gene variants. Treatment intervals are indicated with horizontal bars. In details: CB‐103 (day ‐20 to day +28, day +36 to day +39 and day +64 to day +89); Ponatinib (day +14 to day +27 and from day +57 forward).

Table S1. The patient's prior treatment according to the Group for Research on Adult Acute Lymphoblastic Leukemia (GRAALL) 2014/T protocol and in the setting of relapsed/remitting disease.
