# Supplementary material for: CB‐103: A novel CSL‐NICD inhibitor for the treatment of NOTCH‐driven T‐cell acute lymphoblastic leukemia: A case report of complete clinical response in a patient with relapsed and refractory T‐ALL
Source: EJHaem. 2022 Jun 16;3(3):1009–12. doi: 10.1002/jha2.510 (PMC9421963; doi:10.1002/jha2.510)
Supplement: Supplementary file 1 — Supplement 1_ Methods [file JHA2-3-1009-s004.docx]

**Supplement 1. Method description**

**Sequencing of leukemia cells**

Amplification of target sequences and parallel sequencing (next-generation sequencing) on the Ion Torrent S5 platform using the Ion Torrent Oncomine™ Custom Lymphoma Panel to detect genetic alterations at the DNA level. Data analysis using Torrent Suite™ and Ion Reporter™ Software 5.10.

Technical details of the panel (Custom Lymphoma Panel):

68 genes covered, including 20 genes with complete coverage (ATM, B2M, BCL2, BTG1, CDKN2A, EBF1, GNA13, HIST1H1C, IKZF1, KMT2D, MEF2B, MYC, PAX5, PIM1, PRDM1, PTEN, PTPN1, SOCS1, TNFAIP3, TP53) and 48 genes with hotspot coverage (BCL10, BCL2L11, BCL6, BRAF, CALR, CARD11, CCND1, CD79A, CD79B, CELSR2, CREBBP, DNMT3A, EP300, EZH2, FBXW7, FLT3, FOXO1, IDH1, IDH2, IKZF3, IRF4, JAK2, JAK3, KIT, KLHL6, KRAS, MAP2K1, MCL1, MLL3, MTOR, MYD88, NOTCH1, NOTCH2, NRAS, PIK3CA, PIK3CD, PIK3R1, PTPN11, RELN, RHOA, SF3B1, SGK1, STAT3, STAT6, TET2, TLN2, U2AF1, XPO1).

This method is published (Leukemia 2016;30:2385-2395. doi: 10.1038/leu.2016.135. and J Hematol Oncol. 2017;10:70. doi: 10.1186/s13045-017-0438-7) and validated (Leuk Lymphoma. 2018;59:1710-1716).

**Sequencing of blood cells**

Target amplification was performed using the Oncomine Myeloid Research assay (Thermo Fisher Scientific), covering 40 key DNA target genes, 29 driver genes, and an RNA-based fusion panel:

Hotspot genes: ABL1, BRAF, CBL, CSF3R, DNMT3A, FLT3, GATA2, HRAS, IDH1, IDH2, JAK2, KIT, KRAS, MPL, MYD88, NPM1, NRAS, PTPN11, SETBP1, SFRB1, SRSF2, U2AF1, WT1(Hotspot)

Genes with full exon coverage: ASXL1, BCOR, CALR, CEBPA, ETV6, EZH2, IKZF1, NF1, PHF6, PRPF8, RB1, RUNX1, SH2B3, STAG2, TET2, TP53, ZRSR2 (whole exome including splice sites).

Fusion driver genes: ABL1, ALK, BCL2, BRAF, CCND1, CREBBP, EGFR, ETV6, FGFR1, FGFR2, FUS, HMGA2, JAK2, KMT2A, MLL, MECOM, MET, MLLT10, MLLT3, MYBL1, MYH11, NTRK3, NUP214, PDGFRA, PDGFRB, RARA, RBM15, RUNX1, TCF3, TFE3

Sequencing was performed on an Ion Torrent S5 platform. Human Genome Reference is GRCh37/hg19.

**ctDNA sequencing**

Library preparation was performed using 50 ng input material and a kit from Twist Bioscience, with subsequent enrichment for a panel of 766 tumor-associated genes followed by high throughput next-generation sequencing on the Illumina NovaSeq 6000 platform. A proprietary software was used for variant detection (CeGat, Germany).

Known somatic variants identified pre-HSCT were monitored over the study period forcing the call for variants, independently of the allele frequency.

Gene List

AAK1, ABCB1, ABCG2, ABL1, ABL2, ABRAXAS1, ACD, ACVR1, ADGRA2, ADRB1, ADRB2, AIP, AIRE, AJUBA, AKT1, AKT2, AKT3, ALK, ALOX12B, AMER1, ANKRD26, APC, APLNR, APOBEC3A, APOBEC3B, AR, ARAF, ARHGAP35, ARID1A, ARID1B, ARID2, ARID5B, ASXL1, ASXL2, ATM, ATP1A1, ATR, ATRX, AURKA, AURKB, AURKC, AXIN1, AXIN2, AXL, B2M, BAP1, BARD1, BAX, BCHE, BCL10, BCL11A, BCL11B, BCL2, BCL3, BCL6, BCL9, BCL9L, BCOR, BCORL1, BCR, BIRC2, BIRC3, BIRC5, BLM, BMI1, BMPR1A, BRAF, BRCA1, BRCA2, BRD3, BRD4, BRD7, BRIP1, BTK, BUB1B, CALR, CAMK2G, CARD11, CASP8, CBFB, CBL, CBLB, CBLC, CCDC6, CCND1, CCND2, CCND3, CCNE1, CD274, CD79A, CD79B, CD82, CDC73, CDH1, CDH11, CDH2, CDH5, CDK1, CDK12, CDK4, CDK5, CDK6, CDK8, CDKN1A, CDKN1B, CDKN1C, CDKN2A, CDKN2B, CDKN2C, CEBPA, CENPA, CEP57, CFTR, CHD1, CHD2, CHD4, CHEK1, CHEK2, CIC, CIITA, CKS1B, CNKSR1, COL1A1, COMT, COQ2, CREB1, CREBBP, CRKL, CRLF2, CRTC1, CRTC2, CSF1R, CSF3R, CSMD1, CSNK1A1, CTCF, CTLA4, CTNNA1, CTNNB1, CTRC, CUX1, CXCR4, CYLD, CYP1A2, CYP2A7, CYP2B6, CYP2C19, CYP2C8, CYP2C9, CYP2D6, CYP3A4, CYP3A5, CYP4F2, DAXX, DCC, DDB2, DDR1, DDR2, DDX11, DDX3X, DDX41, DEK, DHFR, DICER1, DIS3L2, DNMT1, DNMT3A, DOT1L, DPYD, E2F3, EBP, EED, EFL1, EGFR, EGLN1, EGLN2, EIF1AX, ELAC2, ELF3, EME1, EML4, EMSY, EP300, EPAS1, EPCAM, EPHA2, EPHA3, EPHA4, EPHB4, EPHB6, ERBB2, ERBB3, ERBB4, ERCC1, ERCC2, ERCC3, ERCC4, ERCC5, ERG, ERRFI1, ESR1, ESR2, ETNK1, ETS1, ETV1, ETV4, ETV5, ETV6, EWSR1, EXO1, EXT1, EXT2, EZH1, EZH2, FAN1, FANCA, FANCB, FANCC, FANCD2, FANCE, FANCF, FANCG, FANCI, FANCL, FANCM, FAS, FAT1, FBXO11, FBXW7, FEN1, FES, FGF10, FGF14, FGF19, FGF2, FGF23, FGF3, FGF4, FGF5, FGF6, FGF9, FGFBP1, FGFR1, FGFR2, FGFR3, FGFR4, FH, FLCN, FLI1, FLT1, FLT3, FLT4, FOXA1, FOXA2, FOXE1, FOXL2, FOXO1, FOXO3, FOXP1, FOXQ1, FRK, FRS2, FUBP1, FUS, FYN, G6PD, GALNT12, GATA1, GATA2, GATA3, GATA4, GATA6, GGT1, GLI1, GLI2, GLI3, GNA11, GNA13, GNAQ, GNAS, GNB3, GPC3, GPER1, GREM1, GRIN2A, GRM3, GSK3A, GSK3B, GSTP1, H3-3A, H3-3B, H3C2, HABP2, HCK, HDAC1, HDAC2, HDAC6, HGF, HIF1A, HLA-A, HLA-B, HLA-C, HLA-DPA1, HLA-DPB1, HLA-DQA1, HLA-DQB1, HLA-DRA, HLA-DRB1, HMGA2, HMGCR, HMGN1, HNF1A, HNF1B, HOXB13, HRAS, HSD3B1, HSP90AA1, HSP90AB1, HTR2A, ID3, IDH1, IDH2, IDO1, IFNGR1, IFNGR2, IGF1R, IGF2, IGF2R, IKBKB, IKBKE, IKZF1, IKZF3, IL1B, IL1RN, ING4, INPP4A, INPP4B, INPPL1, INSR, IRF1, IRF2, IRS1, IRS2, IRS4, ITPA, JAK1, JAK2, JAK3, JUN, KAT6A, KDM5A, KDM5C, KDM6A, KDR, KEAP1, KIAA1549, KIF1B, KIT, KLF2, KLF4, KLHL6, KLLN, KMT2A, KMT2B, KMT2C, KMT2D, KNSTRN, KRAS, KSR1, LATS1, LATS2, LCK, LIG4, LIMK2, LRP1B, LRRK2, LTK, LYN, LZTR1, MAD2L2, MAF, MAGI1, MAGI2, MAML1, MAP2K1, MAP2K2, MAP2K3, MAP2K4, MAP2K5, MAP2K6, MAP2K7, MAP3K1, MAP3K13, MAP3K14, MAP3K3, MAP3K4, MAP3K6, MAP3K8, MAPK1, MAPK11, MAPK12, MAPK14, MAPK3, MAX, MBD1, MC1R, MCL1, MDC1, MDH2, MDM2, MDM4, MECOM, MED12, MEF2B, MEN1, MERTK, MET, MGA, MGMT, MITF, MLH1, MLH3, MLLT10, MLLT3, MN1, MPL, MRE11, MS4A1, MSH2, MSH3, MSH4, MSH5, MSH6, MSR1, MST1R, MTAP, MTHFR, MTOR, MT-RNR1, MTRR, MUC1, MUTYH, MXI1, MYB, MYC, MYCL, MYCN, MYD88, MYH11, MYH9, NAT2, NBN, NCOA1, NCOA3, NCOR1, NF1, NF2, NFE2L2, NFKB1, NFKB2, NFKBIA, NFKBIE, NIN, NKX2-1, NLRC5, NOTCH1, NOTCH2, NOTCH3, NOTCH4, NPM1, NQO1, NR1I3, NRAS, NRG1, NRG2, NSD1, NSD2, NSD3, NT5C2, NT5E, NTHL1, NTRK1, NTRK2, NTRK3, NUMA1, NUP98, NUTM1, OPRM1, PAK1, PAK3, PAK4, PAK5, PALB2, PALLD, PARP1, PARP2, PARP4, PAX3, PAX5, PAX7, PBK, PBRM1, PBX1, PDCD1, PDCD1LG2, PDGFA, PDGFB, PDGFC, PDGFD, PDGFRA, PDGFRB, PDIA3, PDK1, PDPK1, PGR, PHF6, PHOX2B, PIGA, PIK3C2A, PIK3C2B, PIK3C2G, PIK3CA, PIK3CB, PIK3CD, PIK3CG, PIK3R1, PIK3R2, PIK3R3, PIM1, PKHD1, PLCG1, PLCG2, PLK1, PML, PMS1, PMS2, POLD1, POLE, POLH, POLQ, POT1, PPM1D, PPP2R1A, PPP2R2A, PRDM1, PREX2, PRKAR1A, PRKCA, PRKCI, PRKD1, PRKDC, PRKN, PRMT5, PRSS1, PSMB1, PSMB10, PSMB2, PSMB5, PSMB8, PSMB9, PSMC3IP, PSME1, PSME2, PSME3, PSPH, PTCH1, PTCH2, PTEN, PTGS2, PTK2, PTK6, PTK7, PTPN11, PTPN12, PTPRC, PTPRD, PTPRS, PTPRT, RABL3, RAC1, RAC2, RAD21, RAD50, RAD51, RAD51B, RAD51C, RAD51D, RAD54B, RAD54L, RAF1, RALGDS, RARA, RASA1, RASAL1, RB1, RBM10, RECQL4, RET, RFC2, RFWD3, RFX5, RFXANK, RFXAP, RHBDF2, RHEB, RHOA, RICTOR, RINT1, RIPK1, RIT1, RNASEL, RNF43, ROS1, RPS20, RPS6KB1, RPS6KB2, RPTOR, RSF1, RUNX1, RYR1, SAMHD1, SAV1, SBDS, SCG5, SDHA, SDHAF2, SDHB, SDHC, SDHD, SEC23B, SERPINB9, SETBP1, SETD2, SETDB1, SF3B1, SGK1, SH2B1, SH2B3, SHH, SIK2, SIN3A, SKP2, SLC19A1, SLC26A3, SLCO1B1, SLIT2, SLX4, SMAD3, SMAD4, SMARCA4, SMARCB1, SMARCD1, SMARCE1, SMC1A, SMC3, SMO, SOCS1, SOX11, SOX2, SOX9, SPEN, SPINK1, SPOP, SPRED1, SPTA1, SRC, SRD5A2, SRGAP1, SRSF2, SSTR1, SSTR2, SSX1, STAG1, STAG2, STAT1, STAT3, STAT5A, STAT5B, STK11, SUFU, SUZ12, SYK, TAF1, TAF15, TAP1, TAP2, TAPBP, TBK1, TBL1XR1, TBX3, TCF3, TCF4, TCF7L2, TCL1A, TEK, TENT5C, TERC, TERF2IP, TERT, TET1, TET2, TFE3, TGFB1, TGFBR2, TLR4, TLX1, TMEM127, TMPRSS2, TNFAIP3, TNFRSF11A, TNFRSF13B, TNFRSF14, TNFRSF8, TNFSF11, TNK2, TOP1, TOP2A, TP53, TP53BP1, TP63, TPMT, TPX2, TRAF2, TRAF3, TRAF5, TRAF6, TRAF7, TRRAP, TSC1, TSC2, TSHR, TTK, TUBB, TYMS, U2AF1, UBE2T, UBR5, UGT1A1, UGT2B15, UGT2B7, UIMC1, UNG, USP34, USP9X, VEGFA, VEGFB, VHL, VKORC1, WRN, WT1, XIAP, XPA, XPC, XPO1, XRCC1, XRCC2, XRCC3, XRCC5, XRCC6, YAP1, YES1, ZFHX3, ZNF217, ZNF703, ZNRF3, ZRSR2

**T-Cell Receptor rearrangement analysis**

The qPCR based analysis was done using two leukemia-specific DNA markers with a quantitative range of 5.0E-4 and a sensitivity of 1.0E-5.

Marker 1: V1J JD1

Marker 2: Vb19 Jb1.6

The analysis was performed according to the EuroMRD guidelines (van der Velden et al., Leukemia, 2007).
