# Supplementary figures and images for: CB‐103: A novel CSL‐NICD inhibitor for the treatment of NOTCH‐driven T‐cell acute lymphoblastic leukemia: A case report of complete clinical response in a patient with relapsed and refractory T‐ALL
Source: EJHaem. 2022 Jun 16;3(3):1009–12. doi: 10.1002/jha2.510 (PMC9421963; doi:10.1002/jha2.510)

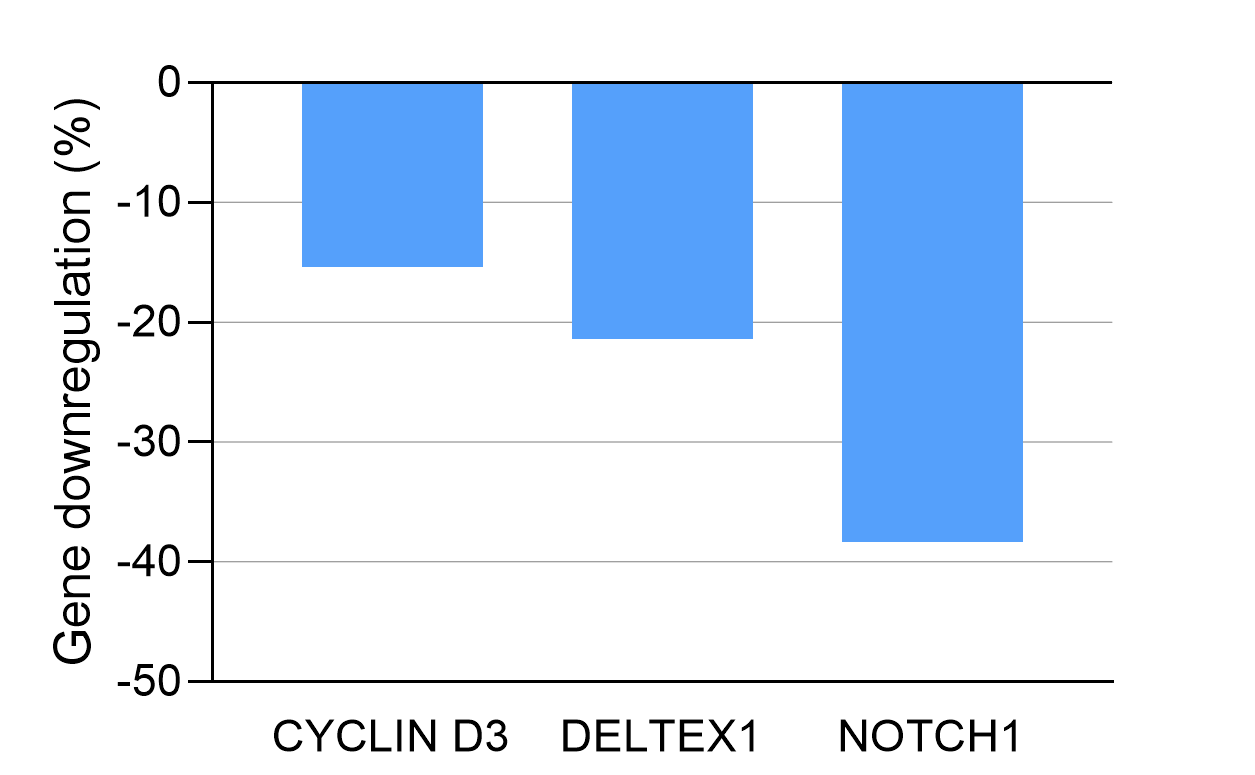

Supplement: Supplementary file 2 — Supplementary Figure 1 – NOTCH pathway target‐gene downregulation 1 hour after CB‐103 administration. Gene expression profiling in peripheral blasts performed using NanoString technology showed downregulation of NOTCH target genes, including Cyclin D3, Deltex‐1 and NOTCH1. [file JHA2-3-1009-s002.png]

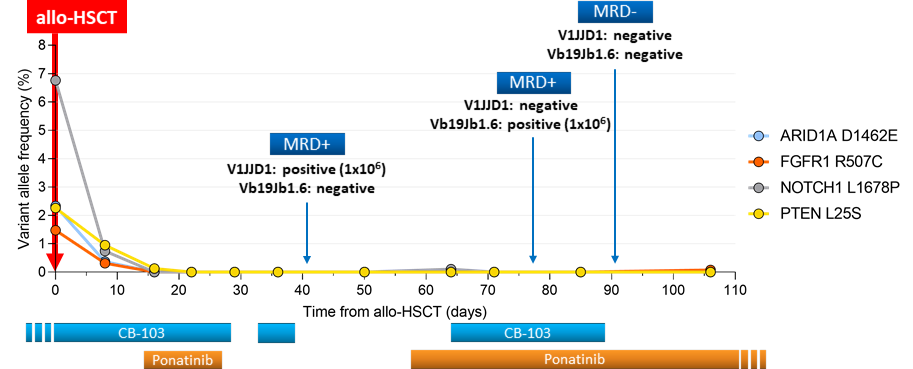

Supplement: Supplementary file 3 — Figure S2 ‐ Presence of T‐ALL molecular markers in liquid biopsies following allogeneic Hematopoietic Stem Cell Transplantation (allo‐HSCT). Next‐generation sequencing of circulating tumour DNA was performed at various timepoints to follow up the identified T‐ALL gene variants. Treatment intervals are indicated with horizontal bars. In details: CB‐103 (day ‐20 to day +28, day +36 to day +39 and day +64 to day +89); Ponatinib (day +14 to day +27 and from day +57 forward). [file JHA2-3-1009-s001.png]
