# Supplementary material for: CB‐103: A novel CSL‐NICD inhibitor for the treatment of NOTCH‐driven T‐cell acute lymphoblastic leukemia: A case report of complete clinical response in a patient with relapsed and refractory T‐ALL
Source: EJHaem. 2022 Jun 16;3(3):1009–12. doi: 10.1002/jha2.510 (PMC9421963; doi:10.1002/jha2.510)
Supplement: Supplementary file 4 — Table S1. The patient's prior treatment according to the Group for Research on Adult Acute Lymphoblastic Leukemia (GRAALL) 2014/T protocol and in the setting of relapsed/remitting disease. [file JHA2-3-1009-s003.docx]

**Supplementary Material**

**Supplementary Table 1**. The patient’s prior treatment according to the Group for Research on Adult Acute Lymphoblastic Leukemia (GRAALL) 2014/T protocol and in the setting of relapsed/remitting disease.

| **Treatment setting** | **Therapeutic agents** |
| --- | --- |
| Induction | prednisone, vincristine, cyclophosphamide, daunorubicin, L-asparaginase, intrathecal methotrexate, cytarabine, dexamethasone |
| First and second consolidation | cytarabine, vincristine, methotrexate, mercaptopurine, cyclophosphamide, etoposide, intrathecal methotrexate, cytarabine, dexamethasone |
| Late intensification | daunorubicin, vincristine, cyclophosphamide, prednisone, L-asparaginase, intrathecal methotrexate, cytarabine, dexamethasone |
| Third consolidation | nelarabine, cyclophosphamide, etoposide, vincristine, MTX, mercaptopurine, intrathecal methotrexate, cytarabine, dexamethasone |
| Salvage treatment | - idarubicin, cytarabine - nelarabine, daratumumab, dasatinib - cyclophosphamide - venetoclax, ponatinib, decitabine |

**Supplementary Figure 1 – NOTCH pathway target-gene downregulation 1 hour after CB-103 administration.** Gene expression profiling in peripheral blasts performed using NanoString technology showed downregulation of NOTCH target genes, including Cyclin D3, Deltex-1 and NOTCH1.

**Supplementary Figure 2 - Presence of T-ALL molecular markers in liquid biopsies following allogeneic Hematopoietic Stem Cell Transplantation (allo-HSCT).** Next-generation sequencing of circulating tumour DNA was performed at various timepoints to follow up the identified T-ALL gene variants. Treatment intervals are indicated with horizontal bars. In details: CB-103 (day -20 to day +28, day +36 to day +39 and day +64 to day +89); Ponatinib (day +14 to day +27 and from day +57 forward).

Bx, biopsy; MRD, minimal residual disease; VAF, variant allele frequency
